# Mitochondrial genome of *Scutiger boulengeri* (Anura, Megophryidae) reveals an exceptionally expanded non-coding region and its phylogenetic position within Megophryidae

**DOI:** 10.3897/zookeys.1293.203467

**Published:** 2026-09-24

**Authors:** Wan-ting Wang, Fu-hao Zhang, Rui Wang, Xu-ze Zhang

**Affiliations:** 1 College of Ecological Environment and Resources, Qinghai Minzu University, Xining, Qinghai, China College of Ecological Environment and Resources, Qinghai Minzu University Xining China; 2 Qinghai Provincial Key Laboratory of High-value Utilization of Characteristic Economic Plants, Xining, Qinghai, China Qinghai Provincial Key Laboratory of High-value Utilization of Characteristic Economic Plants Xining China; 3 Qinghai Provincial Biotechnology and Analytical Test Key Laboratory, Xining, Qinghai, China Qinghai Provincial Biotechnology and Analytical Test Key Laboratory Xining China; 4 College of Pharmacy, Qinghai Minzu University, Xining, Qinghai, China College of Pharmacy, Qinghai Minzu University Xining China

**Keywords:** Megophryidae, mitochondrial genome, phylogenetic relationships, Qinghai-Xizang Plateau, *
Scutiger
*

## Abstract

*Scutiger boulengeri* is an amphibian distributed in high-altitude mountainous environments on the Qinghai-Xizang Plateau and its surrounding regions. However, its mitochondrial genomic characteristics and phylogenetic position remain to be further investigated. In this study, the complete mitochondrial genome of *S. boulengeri* was sequenced and assembled and a preliminary analysis conducted. The results revealed that the mitochondrial genome of *S. boulengeri* is 49,991 bp in length and comprises 13 protein-coding genes (PCGs), 22 tRNA genes, 2 rRNA genes, and an expanded non-coding region encompassing the putative mitochondrial control region. The overall organization is largely consistent with basic characteristics of vertebrate mitochondrial genomes. The overall A+T content of this mitochondrial genome is approximately 69.77%, indicating AT bias. The expanded non-coding region encompassing the putative control region reaches 22,418 bp in length, and large non-coding intergenic spacers were identified in the regions adjacent to *trnW*-*trnF* and *trnP*-*trnQ*. The Ka/Ks values of all 13 PCGs were lower than 1, suggesting that these protein-coding genes as a whole may be subject to purifying selection. Phylogenetic analyses based on *cytb* and concatenated sequences of the 13 PCGs revealed a close relationship between *S. boulengeri* and *Scutiger liupanensis*. Further analysis based on the 13 PCGs dataset suggested that these two species may form a sister group and, together with *Scutiger ningshanensis*, constitute a closely related clade. Divergence time estimation indicated that the divergence between *S. ningshanensis* and the clade comprising *S. boulengeri* and *S. liupanensis* occurred approximately 5.96 MYA, whereas the recent divergence between *S. boulengeri* and *S. liupanensis* occurred approximately 2.10 MYA. These divergence time estimates correspond to the timescale of regional environmental changes during the Pliocene/Pleistocene. In summary, the mitochondrial genome of *S. boulengeri* exhibits characteristics of structural expansion coexisting with functional conservation. This study provides complete mitochondrial genome data for the Tibetan spiny toad and offers fundamental molecular evidence for investigating molecular evolution and conservation genetics of amphibians in the southeastern Qinghai-Xizang Plateau region.

## Introduction

Due to their high sensitivity to environmental disturbances, amphibians face an elevated risk of extinction globally under combined impacts of multiple stressors, including habitat loss, disease transmission, and climate change (Luedtke et al. 2023). As a critical region for modern biodiversity evolution, Qinghai-Xizang Plateau and its surrounding mountain ranges have undergone topographic shaping, climatic change, and ecological remodeling since Cenozoic, which are widely considered to have significantly affected distribution patterns, evolutionary histories, and adaptive traits of regional amphibians (Deng et al. 2020; Spicer et al. 2020; Zhang et al. 2024). As an important component of Asian amphibian fauna, Megophryidae has long attracted academic attention regarding its phylogenetic relationships and genus-level classification owing to its rich species diversity, relatively conservative morphological traits, and complex taxonomic history (Fei and Ye 1990; Chen et al. 2017; Lyu et al. 2023). As a representative high-altitude group within Megophryidae, *Scutiger* is mainly distributed across Qinghai-Xizang Plateau, Himalayas, and Hengduan Mountains, with an intrinsic relationship between its diversification history and regional geo-environmental evolution serving as persistent research focus (Hofmann et al. 2017). Furthermore, previous studies have demonstrated that complex topographic features and historical environmental dynamics of Qinghai-Xizang Plateau may have jointly shaped genetic structures and lineage diversification patterns of amphibians in this region (Zhang et al. 2026).

However, constrained by high morphological conservatism and complex phenotypic polymorphism, taxonomic relationships and phylogenetic lineages within *Scutiger* require further investigation (Fei et al. 1986). As one of widely distributed species within the genus, previous studies have revealed that *Scutiger boulengeri* harbors multiple internal genetic lineages accompanied by niche divergence and morphological differentiation (Lin et al. 2021). Research indicates presence of broad hybrid zone in *S. boulengeri* mediated by precipitation-related environmental factors, where processes such as historical isolation, secondary contact, and persistent gene flow jointly shape its geographic genetic structure and lead to mitonuclear discordance (Lin et al. 2023). Additionally, mitochondrial introgression resulting from historical interspecific hybridization has been reported among closely related high-altitude megophryid species (Chen et al. 2009). Therefore, investigating complete mitochondrial genome of *S. boulengeri* provides essential molecular foundation for further clarifying its phylogenetic relationships and mitogenomic structural characteristics.

Animal mitochondrial genomes are typically compact, mostly circular molecules of ~ 16 kb with relatively conservative gene organization, and their structural features provide critical insights into amphibian genomic evolutionary patterns and phylogenetic relationships (Zardoya 2020). However, structural variations such as control region variations, repetitive sequence expansions, and localized gene rearrangements have been reported in Megophryidae and related amphibian taxa (Zhang et al. 2021; Zhou et al. 2023; Shan et al. 2025). Because repetitive sequences tend to cause incomplete short-read assemblies, long-read sequencing exhibits distinct advantages in resolving ultra-long non-coding regions, including control region, and complex repetitive regions in amphibians (Asaeda et al. 2023). Thus, employing hybrid assembly strategy combining short and long reads to obtain complete mitochondrial genome of *S. boulengeri* effectively ensures assembly integrity across complex regions, thereby facilitating precise characterization of its non-coding region features.

In this study, we employed a hybrid sequencing strategy combining Illumina and Oxford Nanopore technologies to determine the complete mitochondrial genome of *S. boulengeri*. Based on these data, we analyzed its mitochondrial gene composition, base composition, codon usage bias, and selective pressures, with a specific focus on characterizing the structural organization of the complex non-coding region – encompassing the putative mitochondrial control region – and its adjacent non-coding intergenic spacers. Furthermore, we reconstructed phylogenetic trees based on *cytb* sequences and concatenated sequences of 13 mitochondrial protein-coding genes (PCGs), respectively, to clarify the phylogenetic position of this species at the mitochondrial level and estimate divergence times among closely related lineages within *Scutiger*. These findings enhance our understanding of the structural evolutionary characteristics of the mitogenome in *S. boulengeri*, while providing essential baseline data for future phylogenetic and comparative mitogenomic studies of Megophryidae.

## Materials and methods

### Sample collection and DNA extraction

In August 2024, an adult female specimen of *Scutiger boulengeri* was collected from Yaertang Township, Guoluo Tibetan Autonomous Prefecture, Qinghai Province, China (32°46'N, 100°49'E; elevation ca 3,335 m; Fig. 1). For subsequent genomic DNA extraction, fresh muscle tissue was preserved in 95% ethanol and stored in an ultra-low temperature freezer at −80 °C. The tissue specimen (voucher number SB250820) was deposited in the College of Eco-Environment and Resources, Qinghai Minzu University. Species identification was verified using *Fauna of China* and the *Color Atlas of Chinese Amphibians and Their Distribution* (Fei et al. 2009; Fei 2012). The specimens exhibited external morphological characteristics consistent with published descriptions, including a blunt, rounded snout, the absence of a distinct tympanum, rough dorsal skin bearing granular protuberances, rounded and blunt toe tips, and a ventral surface covered with wart-like granules but lacking spines. All experimental procedures were reviewed and approved by the Science and Technology Ethics Review Committee of Qinghai Minzu University and were conducted in accordance with relevant guidelines for laboratory animal welfare.

**Figure 1.**
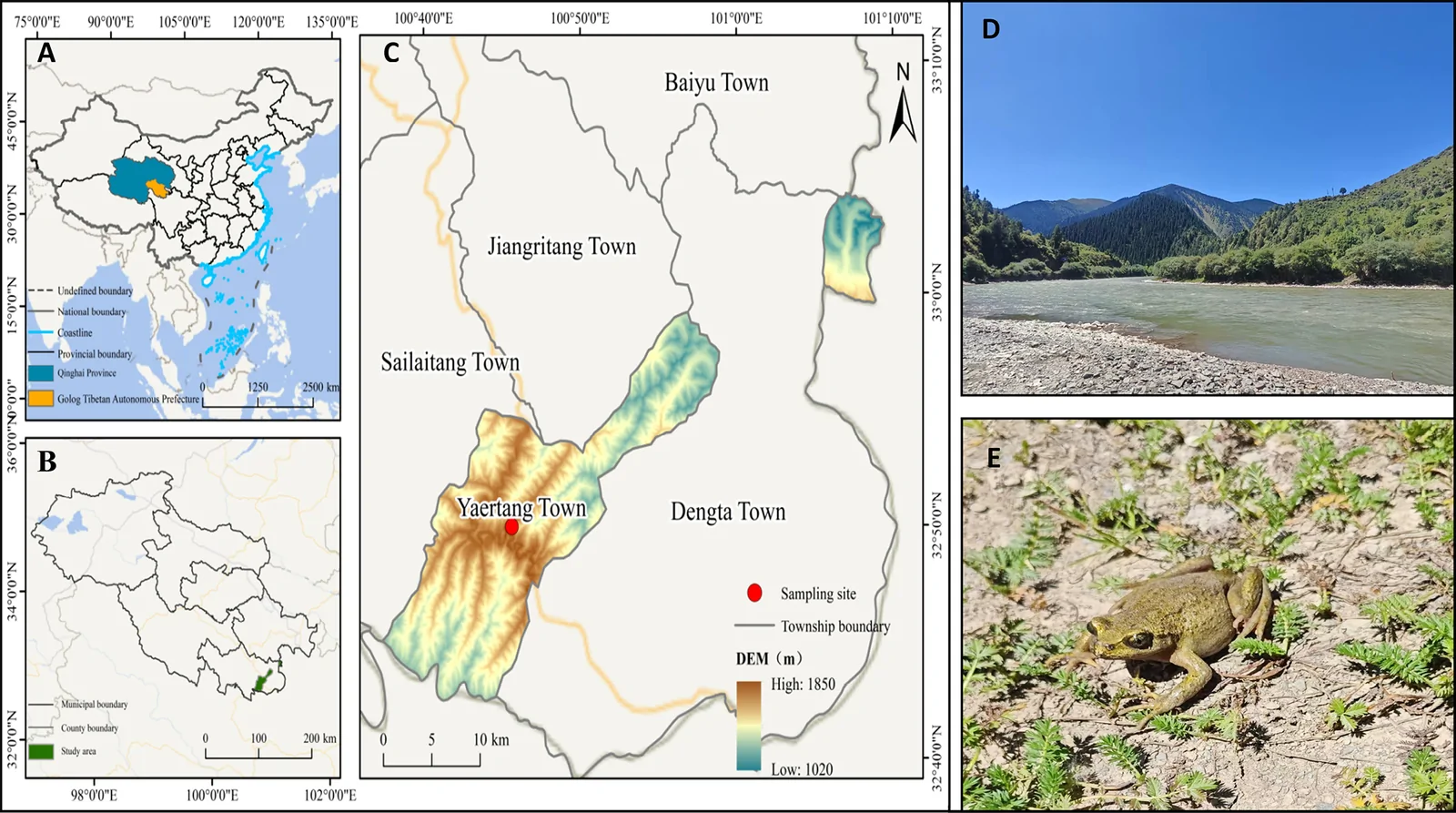
Sampling location, habitat, and photo of live adult *Scutiger boulengeri* on the Qinghai-Xizang Plateau. **A**. Location of Qinghai Province and Guoluo Tibetan Autonomous Prefecture within China; **B**. Location of study area within Guoluo Tibetan Autonomous Prefecture, Qinghai Province; **C**. Sampling location in Yaertang Township, Baima County, Guoluo Tibetan Autonomous Prefecture, Qinghai Province, along with surrounding townships and topographic distribution, where red dot indicates sampling site; **D**. Habitat at sampling site; **E**. Photograph of *S. boulengeri*.

### DNA extraction and sequencing

Total genomic DNA was extracted from samples using a standard phenol/chloroform method (Green and Sambrook 2017). The integrity of the extracted DNA was evaluated via 1% agarose gel electrophoresis, while its purity and concentration were assessed using a NanoPhotometer spectrophotometer (IMPLEN, USA), a Qubit 2.0 Fluorometer (Life Technologies, USA), and an Agilent Bioanalyzer 2100 (Agilent Technologies, USA), respectively. To accurately resolve the complex regions of the mitochondrial genome, a combined sequencing strategy utilizing Illumina short reads and Oxford Nanopore long reads was employed. Specifically, short-read data were generated on the Illumina NovaSeq platform via PE150 paired-end sequencing mode, yielding 24,255,804 clean reads with a total sequencing volume of 7.33 Gb. Concurrently, long-read data were obtained using the Oxford Nanopore PromethION platform, producing 858,550 reads with a sequencing volume of 7.95 Gb, average read length of 9,255 bp, and read N50 of 13,009 bp.

### Assembly and annotation

The raw sequencing reads were initially subjected to quality trimming and filtering using fastp v. 0.20.0 (Chen et al. 2018). Subsequently, mitochondrial-origin reads were identified by aligning the processed reads with Bowtie2 v. 2.2.4 in very-sensitive-local mode (Langmead and Salzberg 2012). Preliminary assembly of the mitochondrial genome was performed using SPAdes v. 3.10.1 (Bankevich et al. 2012). To address potential assembly gaps caused by regions of high AT content or complex repeats, long-read data generated by Oxford Nanopore technology were aligned using Minimap2 v. 2.15-r905 (Li 2018), followed by assembly and error correction with Canu v. 2.3 (Koren et al. 2017). The resulting assembly was further polished by integrating short-read data using NextPolish v. 1.4.1 (Hu et al. 2020). The sequencing reads were mapped back to the final assembled mitochondrial genome, yielding 57,059 mapped mitochondrial reads and an average sequencing depth of 368.03×. Annotation of the mitochondrial genome was conducted with MITOS2 (Bernt et al. 2013), and tRNA genes along with their secondary structures were manually verified. Base composition, AT/GC skew, and relative synonymous codon usage (RSCU) were calculated using a custom Perl script. A circular representation of the mitochondrial genome was generated with OGDRAW v. 1.3.1 (Greiner et al. 2019). Fully annotated mitochondrial genome of *S. boulengeri* was submitted to the CNCB database in TBL format under accession number C_AA451531.

### Phylogenetic analysis

To explore the mitochondrial phylogenetic position of *S. boulengeri* within *Scutiger* and Megophryidae, two datasets were constructed: a single-gene *cytb* sequence dataset for *Scutiger* and related taxa (Table 1), and a concatenated sequence dataset of 13 mitochondrial protein-coding genes (13 PCGs) (Table 2). The *cytb* dataset was used to compare maternal relationships of *S. boulengeri* within *Scutiger*, whereas the 13 PCGs dataset was utilized to evaluate its phylogenetic position among Megophryidae-related taxa at the mitochondrial genome scale. The *cytb* dataset was rooted with *Rana kukunoris* as outgroup, while the 13 PCGs dataset was rooted with *R. kukunoris* and *Nanorana pleskei* as outgroups. All sequences were aligned using MAFFT v. 7.505 (Katoh and Standley 2013), and low-quality or poorly conserved aligned regions were removed using trimAl v. 1.4 (Capella-Gutiérrez et al. 2009) and Gblocks v. 0.91b (Talavera and Castresana 2007). The concatenated 13 PCGs matrix comprised 30 taxa and 11,295 sites, and was divided into 39 initial partitions based on gene and codon position. maximum likelihood (ML) analysis was performed in IQ-TREE v. 2.2.0 (Minh et al. 2020), with the best-fit nucleotide substitution models for each partition selected by ModelFinder (Kalyaanamoorthy et al. 2017), and node support evaluated via 1,000 ultrafast bootstrap replicates (Kalyaanamoorthy et al. 2017; Minh et al. 2020). Bayesian inference (BI) was conducted using MrBayes v. 3.2.7a (Ronquist et al. 2012). Partition schemes and substitution models were selected by PartitionFinder v. 2.1.1 under AICc criterion and greedy search strategy (Lanfear et al. 2017). Two independent runs were executed, each with four Markov chains for 3,000,000 generations, and initial 25% of the sampled trees were discarded as burn-in. Finally, analytical convergence was assessed based on effective sample size (ESS > 200) and topological stability.

**Table 1.** Taxonomic information, GenBank accession numbers, sequence lengths, and sample names of taxa included in the c*ytb* phylogenetic dataset. *Rana kukunoris* was designated as the outgroup.

**Family**	**Species**	**Accession number**	**Sequence length**	**Reference**
Megophryidae	***Scutiger boulengeri***	**C_AA451531**	**1141 bp**	**This study**
	*Scutiger boulengeri*	KJ082072.1	1009 bp	Meng et al. (2014)
	*Scutiger boulengeri*	OM505282.1	991 bp	Lin et al. (2023)
	*Scutiger boulengeri*	FJ463049.1	1005 bp	Li et al. (2009)
	*Scutiger boulengeri*	FJ463124.1	1035 bp	Li et al. (2009)
	*Scutiger boulengeri*	MW600729.1	971 bp	Lin et al. (2021)
	*Scutiger boulengeri*	FJ463147.1	1038 bp	Li et al. (2009)
	*Scutiger boulengeri*	MW133438.1	939 bp	Che et al. (2020)
	*Scutiger boulengeri*	MK970631.1	889 bp	Khatiwada et al. (2019)
	*Scutiger boulengeri*	PP349710.1	985 bp	Hofmann et al. (2024)
	*Scutiger ningshanensis*	KF757391.1	1009 bp	Meng et al. (2014)
	*Scutiger liupanensis*	JX533806.1	947 bp	GenBank
	*Scutiger chintingensis*	KY310930.1	985 bp	Hofmann et al. (2017)
	*Scutiger gongshanensis*	MW133458.1	614 bp	Che et al. (2020)
	*Scutiger mammatus*	OM505279.1	991 bp	Lin et al. (2023)
	*Scutiger nepalensis*	KY310952.1	985 bp	Hofmann et al. (2017)
	*Scutiger occidentalis*	MZ126691.1	985 bp	Hofmann et al. (2021)
	*Scutiger sikimmensis*	KY310971.1	985 bp	Hofmann et al. (2017)
	*Scutiger tuberculatus*	MW133413.1	939 bp	Che et al. (2020)
	*Scutiger jiulongensis*	MW133417.1	939 bp	Che et al. (2020)
	*Scutiger muliensis*	MW133421.1	939 bp	Che et al. (2020)
	*Scutiger nyingchiensis*	OM505293.1	991 bp	Lin et al. (2023)
	*Scutiger spinosus*	MW133428.1	939 bp	Che et al. (2020)
	*Scutiger wuguanfui*	MW133435.1	939 bp	Che et al. (2020)
	*Scutiger ghunsa*	MK970628.1	889 bp	Khatiwada et al. (2019)
	*Scutiger glandulatus*	OM505267.1	991 bp	Lin et al. (2023)
Ranidae	*Rana kukunoris*	KM211951.1	800 bp	Qi et al. (2014)

Note: With the exception of species newly sequenced in this study (in bold), all other *cytb* sequences for genus *Scutiger* were retrieved from GenBank. When multiple *cytb* sequence records were available for a single taxon, the sequence containing the longest *cytb* region was selected for phylogenetic analysis.

**Table 2.** Taxonomic information, GenBank accession numbers, mitochondrial genome lengths, and sample names of taxa included in the concatenated dataset of 13 mitochondrial protein-coding genes. *Rana kukunoris* and *Nanorana pleskei* were designated as outgroups.

**Family**	**Species**	**Accession number**	**Sequence length**	**Reference**
Megophryidae	***Scutiger boulengeri***	**C_AA451531**	**49991 bp**	**This study**
	*Scutiger liupanensis*	KX352261.1	16890 bp	Fan (2016)
	*Scutiger ningshanensis*	NC_031426.1	17265 bp	Shan et al. (2025)
	*Megophrys carinense*	JX564854.1	15271 bp	Zhang et al. (2013)
	*Megophrys gigantica*	MZ364157.1	18259 bp	GenBank
	*Megophrys omeimontis*	KP728257.1	17013 bp	Liu et al. (2016)
	*Megophrys shapingensis*	NC_018785.1	17631 bp	Xiang et al. (2013)
	*Megophrys* sp. FH-2015	KT601071.1	17866 bp	GenBank
	*Megophrys* sp. HW-2022	NC_060625.1	17263 bp	GenBank
	*Oreolalax rhodostigmatus*	ON646613.1	18854 bp	GenBank
	*Oreolalax omeimontis*	NC_049862.1	17675 bp	Wang et al. (2022)
	*Oreolalax major*	MN803320.1	17786 bp	Huang et al. (2020)
	*Oreolalax lichuanensis*	NC_030627.1	17702 bp	Liang et al. (2016)
	*Oreolalax multipunctatus*	NC_037382.1	17358 bp	Li et al. (2018)
	*Oreolalax schmidti*	NC_056343.1	18481 bp	Bao et al. (2020)
	*Oreolalax xiangchengensis*	MH727696.1	17110 bp	Li et al. (2018)
	*Panophrys tuberogranulata*	OQ830573.1	16841 bp	Xiang et al. (2024)
	*Leptobrachium boringii*	NC_024427.1	17085 bp	Xu et al. (2016)
	*Leptobrachium liui*	NC_057468.1	17190 bp	Xiang et al. (2021)
	*Leptobrachium leishanense*	KU760082.1	17485 bp	Liang et al. (2016)
	*Leptobrachella pelodytoides*	JX564874.1	14682 bp	Zhang et al. (2013)
	*Leptobrachella oshanensis*	NC_020610.1	17747 bp	Xiang et al. (2013)
	*Leptobrachella alpina*	NC_088425.1	17763 bp	Shu et al. (2021)
	*Boulenophrys baishanzuensis*	NC_081000.1	17040 bp	Wu et al. (2024)
	*Boulenophrys sangzhiensis*	OQ830572.1	16950 bp	Xiang et al. (2024)
	*Boulenophrys boettgeri*	OR529440.1	16597 bp	Wang et al. (2024)
	*Boulenophrys kuatunensis*	OR522721.1	17921 bp	Wang et al. (2024)
	*Boulenophrys spinata*	ON646614.1	16024 bp	GenBank
Ranidae	*Rana kukunoris*	MZ043820.1	21913 bp	Zhang et al. (2023)
	*Nanorana pleskei*	HQ324232.1	17660 bp	Chen et al. (2011)

### Analysis of Ka/Ks selection pressure

We used PhyloSuite v. 1.2.3 to extract the coding sequences of the 13 PCGs from target species *S. boulengeri* and comparative taxa *S. ningshanensis*, *S. liupanensis*, *R. kukunoris*, and *N. pleskei* (Zhang et al. 2020), followed by multiple sequence alignment using MAFFT v. 7.505. The resulting alignments were subsequently converted into AXT format in batch using a Perl script and imported into Ka/Ks_Calculator 3.0 (Zhang 2022). The nonsynonymous substitution rate (Ka) and synonymous substitution rate (Ks) were estimated under the Yang-Nielsen (YN) model (Yang and Nielsen 2000), and the Ka/Ks ratio (ω = Ka/Ks) was calculated to evaluate selective pressure acting on protein-coding genes. Specifically, ω > 1, ω = 1, and ω < 1 indicate positive selection, neutral evolution, and purifying selection, respectively.

### Divergence time estimation

Based on the concatenated 13 PCGs matrix, Bayesian divergence time estimation was performed using the MCMCTree program implemented in PAML v. 4.10 (Yang 2007). The analysis constrained the optimal tree topology derived from 13 PCGs phylogenetic reconstruction and employed the GTR nucleotide substitution model alongside the approximate likelihood method. Specifically, the gradient and Hessian matrix of the branch lengths were calculated using baseml (usedata = 3) and subsequently incorporated into MCMCTree for time estimation (usedata = 2). A correlated rates model (clock = 3) was adopted for the evolutionary clock. The substitution rate prior was set to rgene_gamma = G(2, 3.4, 1), the rate drift prior to sigma2_gamma = G(1, 4.5, 1), and a birth-death process (BDparas = 1 1 0.1) was employed for node time prior distributions (He et al. 2026). For secondary time calibrations, uniform priors with soft bounds were applied, with upper and lower tail probabilities both set to 2.5% Calibration nodes were defined according to (Feng et al. 2017): divergence between Ranidae and Dicroglossidae (Node 1: 162.5–182.6 Mya), divergence between *Rana* and *Nanorana* (Node 2: 56.0–66.4 Mya), divergence between *Scutiger* and the remaining clades (Node 3: 15.2–22.9 Mya), and divergence between *Leptobrachium* and *Oreolalax* (Node 4: 12.1–19.6 Mya). Two independent MCMC runs were executed using different random seeds. For each run, the initial 1,000,000 generations were discarded as burn-in, followed by 10,000 samples drawn for posterior distribution estimation. Convergence was evaluated by calculating the correlation coefficient of node ages between the two runs (r = 1.0000). Prior sensitivity was tested by conducting a prior-only analysis without sequence data (usedata = 0). Analytical convergence was assessed using Tracer v. 1.7.2 (Rambaut et al. 2018), ensuring that effective sample sizes (ESS) for all parameters were > 200.

## Results

### General characteristics of the mitochondrial genome of *Scutiger boulengeri*

The complete mitochondrial genome of *S. boulengeri* is a typical circular double-stranded DNA molecule with a total length of 49,991 bp. This mitochondrial genome contains 37 genes, including 13 protein-coding genes (PCGs), 22 tRNA genes, 2 rRNA genes, and an expanded non-coding region encompassing the putative mitochondrial control region (Fig. 2). Among these 37 genes, nine genes, including *trnA*, *trnN*, *trnC*, *trnY*, *trnS2*, *nad6*, *trnE*, *trnP*, and *trnQ*, are encoded on the light strand (L strand), whereas the remaining genes are encoded on the heavy strand (H strand). The overall gene arrangement of this mitochondrial genome is similar to that of most vertebrate mitochondrial genomes; however, localized atypical arrangements of tRNA genes were observed in regions adjacent to the control region. Comparison of the mitochondrial genome structures of the closely related species *Scutiger liupanensis* and *Scutiger ningshanensis* (Fig. 3) revealed that the three *Scutiger* species exhibit a high degree of conservation in the arrangement of most protein-coding genes and rRNA genes. However, *S. boulengeri* differs from the other two species in terms of non-coding region length and the positions of specific tRNA genes. *Scutiger boulengeri* contains an expanded non-coding region encompassing the putative control region, with a length of 22,418 bp, whereas the corresponding regions in *S. liupanensis* and *S. ningshanensis* are only 1,588 bp and 1,480 bp in length, respectively. Regarding gene arrangement, *S. boulengeri* exhibits translocation events involving the *trnF* and *trnQ* genes. Specifically, the *trnF* gene is translocated between the *trnW* and *trnI* genes. Meanwhile, the *trnQ* gene, which is located between the *trnI* and *trnM* genes in *S. ningshanensis*, is located between the *trnP* gene and the aforementioned expanded putative control region in *S. boulengeri*. Sequence structure analysis revealed nine gene overlap regions in this mitochondrial genome, with a total length of 27 bp. In addition, 16 intergenic spacer regions were identified, with a combined length of 11,239 bp. The largest intergenic spacer was located between *trnW* and *trnF*, with a length of 7,120 bp (Table 4). Regarding nucleotide composition, the mitochondrial genome exhibited nucleotide frequencies of A = 31.69%, T = 38.07%, G = 11.49%, and C = 18.75%. The A+T content (69.77%) was higher than the G+C content (30.23%) (Table 3). The nucleotide composition followed the order T > A > C > G, and the AT-skew and GC-skew values were –0.091 and –0.240, respectively, indicating an overall bias toward T and C nucleotides in the mitochondrial genome.

**Figure 2.**
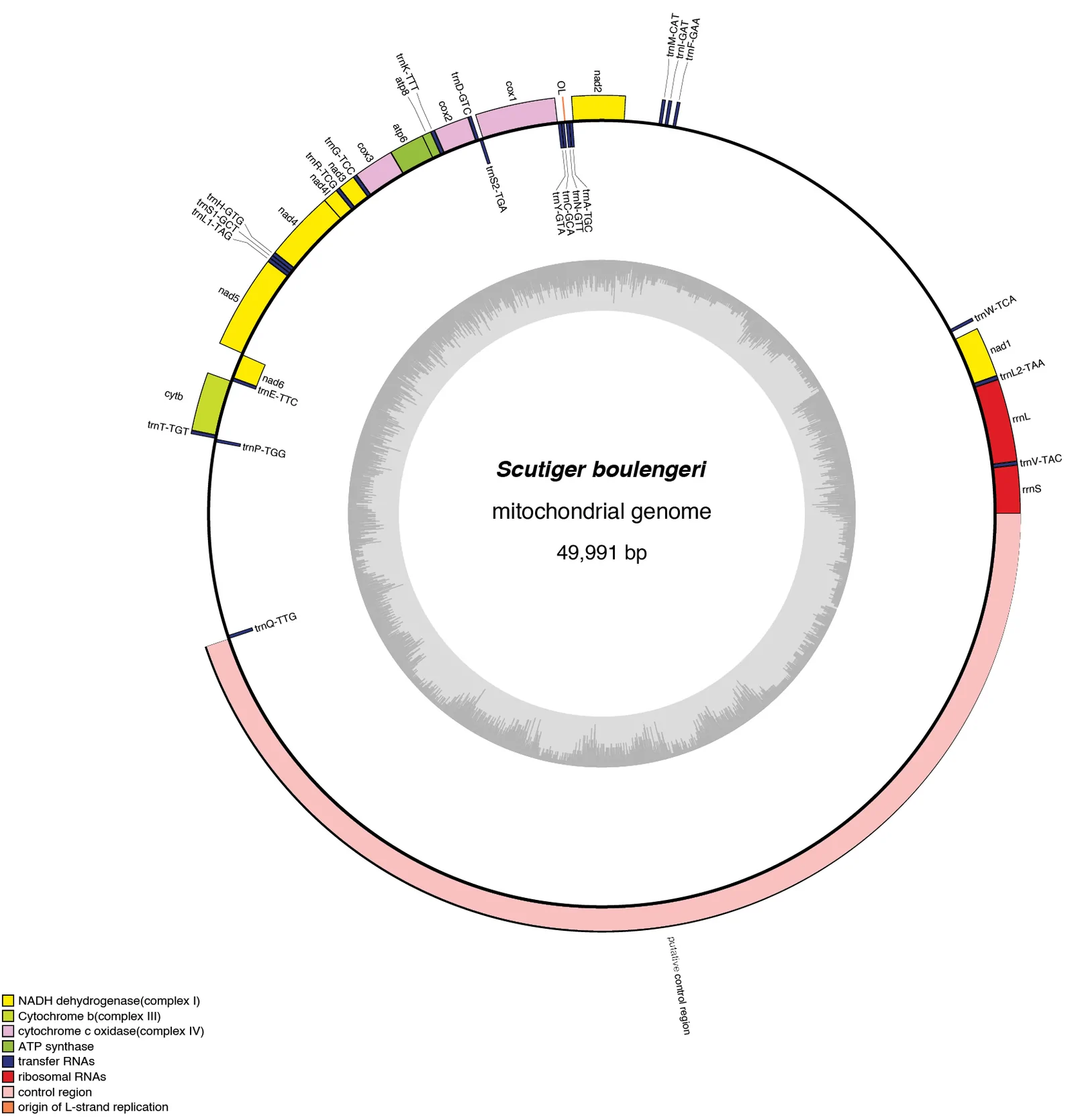
Circular map of the complete mitochondrial genome of *Scutiger boulengeri*. Genes encoded on the heavy and light strands are indicated by the outer and inner rings, respectively, while GC content is represented by the inner gray circle. Significant expansion of non-coding regions is visually evident, particularly the expanded non-coding region encompassing the putative mitochondrial control region.

**Figure 3.**
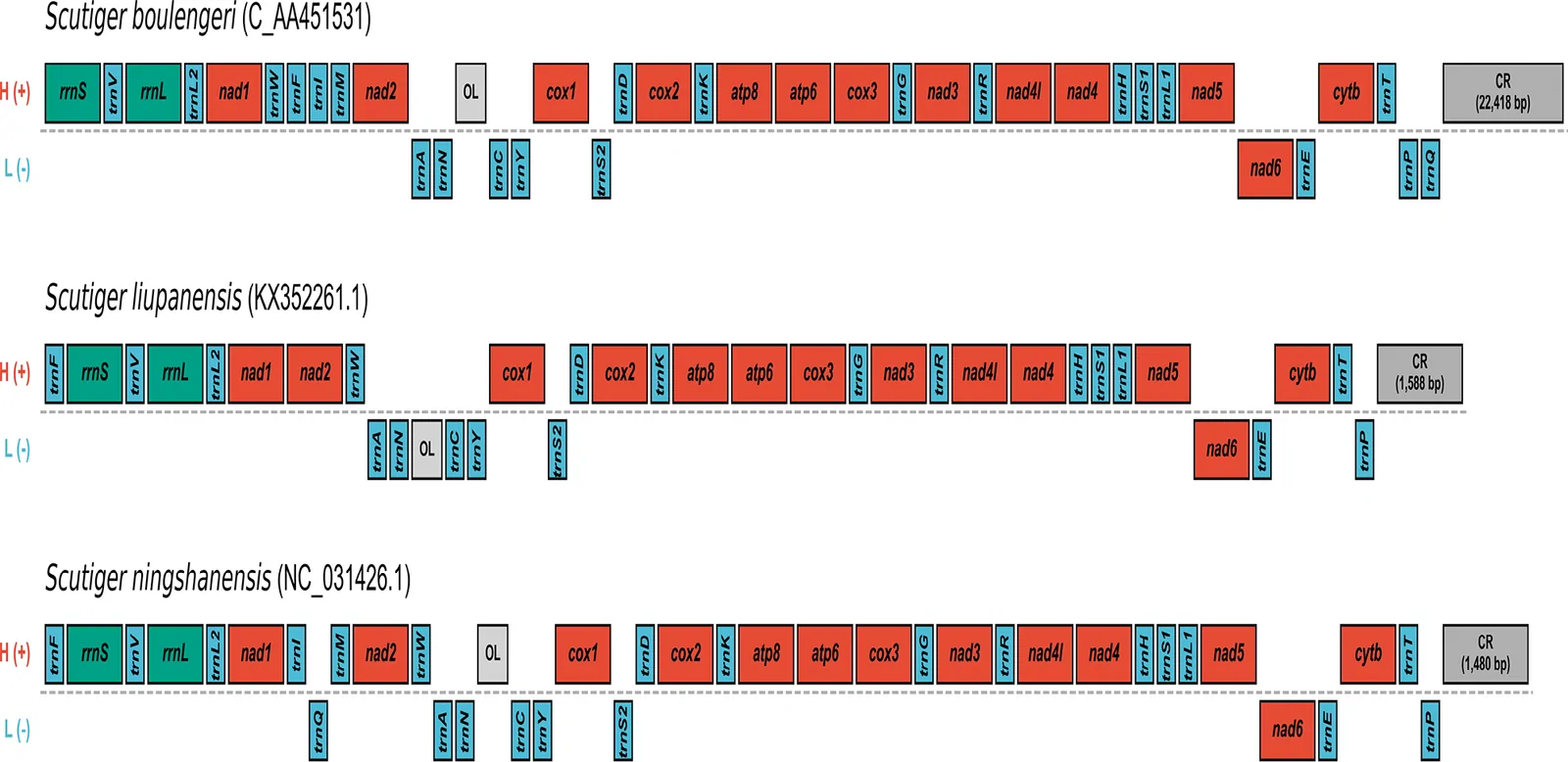
Comparison of mitochondrial genome organization among three species of *Scutiger*. The mitochondrial genome organizations of *Scutiger boulengeri* (C_AA451531), *Scutiger liupanensis* (KX352261.1), and *Scutiger ningshanensis* (NC_031426.1) were compared. Notably, the publicly available annotation of the reference mitochondrial genome of *S. liupanensis* (KX352261.1) does not include the *trnL*, *trnQ*, or *trnM* genes. In the figure, red, blue, and green boxes indicate protein-coding genes (PCGs), transfer RNA (tRNA) genes, and ribosomal RNA (rRNA) genes, respectively, whereas gray boxes denote the origin of light-strand replication (OL) and the control region (CR). Overall, mitochondrial gene order is highly conserved among the three species. However, *S. boulengeri* possesses a markedly expanded control region (22,418 bp) and exhibits differences in the arrangement of several tRNA genes.

**Table 3.** Nucleotide composition, A+T content, G+C content, and AT/GC skew values of the complete mitochondrial genome and major genomic regions of *Scutiger boulengeri*.

***Scutiger boulengeri***	**Size (bp)**	**A%**	**T%**	**G%**	**C%**	**A+T%**	**G+C%**	**AT-skew**	**GC-skew**
Mitogenome	49991	31.69	38.07	11.49	18.75	69.77	30.23	-0.091	-0.24
PCGs	11399	25.8	34.8	14.83	24.56	60.6	39.4	-0.149	-0.247
tRNAs	1540	29.29	28.31	21.88	20.52	57.6	42.4	0.017	0.032
rRNAs	2530	33.12	26.21	17.83	22.85	59.33	40.67	0.117	-0.123
putative control region	22418	34.04	40.66	8.39	16.91	74.7	25.3	-0.089	-0.337

**Table 4.** Organization of the mitochondrial genome of *Scutiger boulengeri*, including gene positions, strand orientation, gene lengths, intergenic spacers, overlapping regions, anticodons, and start and stop codons.

**Gene**	**Direction**	**Location (bp)**	**Size (bp)**	**Intergenic length**	**Anticodon**	**Start codon**	**Stop codon**
*rrnS*	+	1–930	930	0	-	-	-
*trnV*	+	931-1000	70	0	TAC	-	-
*rrnL*	+	1001-2600	1600	0	-	-	-
*trnL2*	+	2601-2675	75	0	TAA	-	-
*nad1*	+	2676-3653	978	0	-	ATG	TAA
*trnW*	+	3806-3874	69	152	TCA	-	-
*trnF*	+	10995-11061	67	7120	GAA	-	-
*trnI*	+	11155–11225	71	93	GAT	-	-
*trnM*	+	11277-11346	70	51	CAT	-	-
*nad2*	+	12039-13082	1044	692	-	ATG	TAA
*trnA*	-	13086-13155	70	3	TGC	-	-
*trnN*	-	13156-13228	73	0	GTT	-	-
OL(rep_origin)	+	13231-13257	27	2	-	-	-
*trnC*	-	13257-13319	63	-1	GCA	-	-
*trnY*	-	13320-13389	70	0	GTA	-	-
*cox1*	+	13391-14942	1552	1	-	GTG	T--
*trnS2*	-	14944-15014	71	1	TGA	-	-
*trnD*	+	15019-15086	68	4	GTC	-	-
*cox2*	+	15088-15775	688	1	-	ATG	T--
*trnK*	+	15776-15849	74	0	TTT	-	-
*atp8*	+	15850-16029	180	0	-	GTG	TAA
*atp6*	+	16020-16702	683	-10	-	ATG	TA-
*cox3*	+	16702-17485	784	-1	-	ATG	T--
*trnG*	+	17486-17554	69	0	TCC	-	-
*nad3*	+	17555-17897	343	0	-	ATG	T--
*trnR*	+	17898-17966	69	0	TCG	-	-
*nad4l*	+	17967-18263	297	0	-	ATG	TAA
*nad4*	+	18257-19634	1378	-7	-	ATG	T--
*trnH*	+	19635-19703	69	0	GTG	-	-
*trnS1*	+	19704-19770	67	0	GCT	-	-
*trnL1*	+	19771-19841	71	0	TAG	-	-
*nad5*	+	19842-21662	1821	0	-	ATG	TAG
*nad6*	-	21678-22187	510	15	-	GTG	AGG
*trnE*	-	22188-22256	69	0	TTC	-	-
*cytb*	+	22259-23399	1141	2	-	ATG	T--
*trnT*	+	23400-23469	70	0	TGT	-	-
*trnP*	-	23472-23540	69	2	TGG	-	-
*trnQ*	-	27498-27573	76	3957	TTG	-	-
putative control region	+	27574-49991	22418	0	-	-	-

Note: “–” indicates genes encoded on the light (L) strand, whereas “+” indicates genes encoded on the heavy (H) strand. Negative values of intergenic spacer length indicate overlaps between adjacent genes.

### Analysis of protein-coding genes and codon usage bias

The 13 protein-coding genes (PCGs) of *S. boulengeri* mitochondrial genome have a combined length of 11,399 bp, accounting for 22.8% of the total genome length. The nucleotide composition of the PCG region was A = 25.8%, T = 34.8%, G = 14.83%, and C = 24.56%, with AT-skew and GC-skew values of –0.149 and –0.247, respectively. Most PCGs initiated with the conventional start codon ATG, whereas only *cox1*, *atp8*, and *nad6* used GTG as the start codon. With respect to termination codons, *cox1*, *cox2*, *cox3*, *nad3*, *nad4*, and c*ytb* terminated with the incomplete stop codon T--, while *atp6* used the incomplete stop codon TA-. Most of the remaining genes terminated with TAA or TAG, whereas nad6 terminated with AGG (Table 4). Sequence statistics showed that the 13 PCGs comprised a total of 2,303 codons. RSCU analysis revealed substantial variation in codon usage frequencies among synonymous codons (Table 5; Fig. 4). Most frequently used codons ended with A or T, suggesting that codon usage bias is associated with the AT-rich composition of the mitochondrial genome.

**Figure 4.**
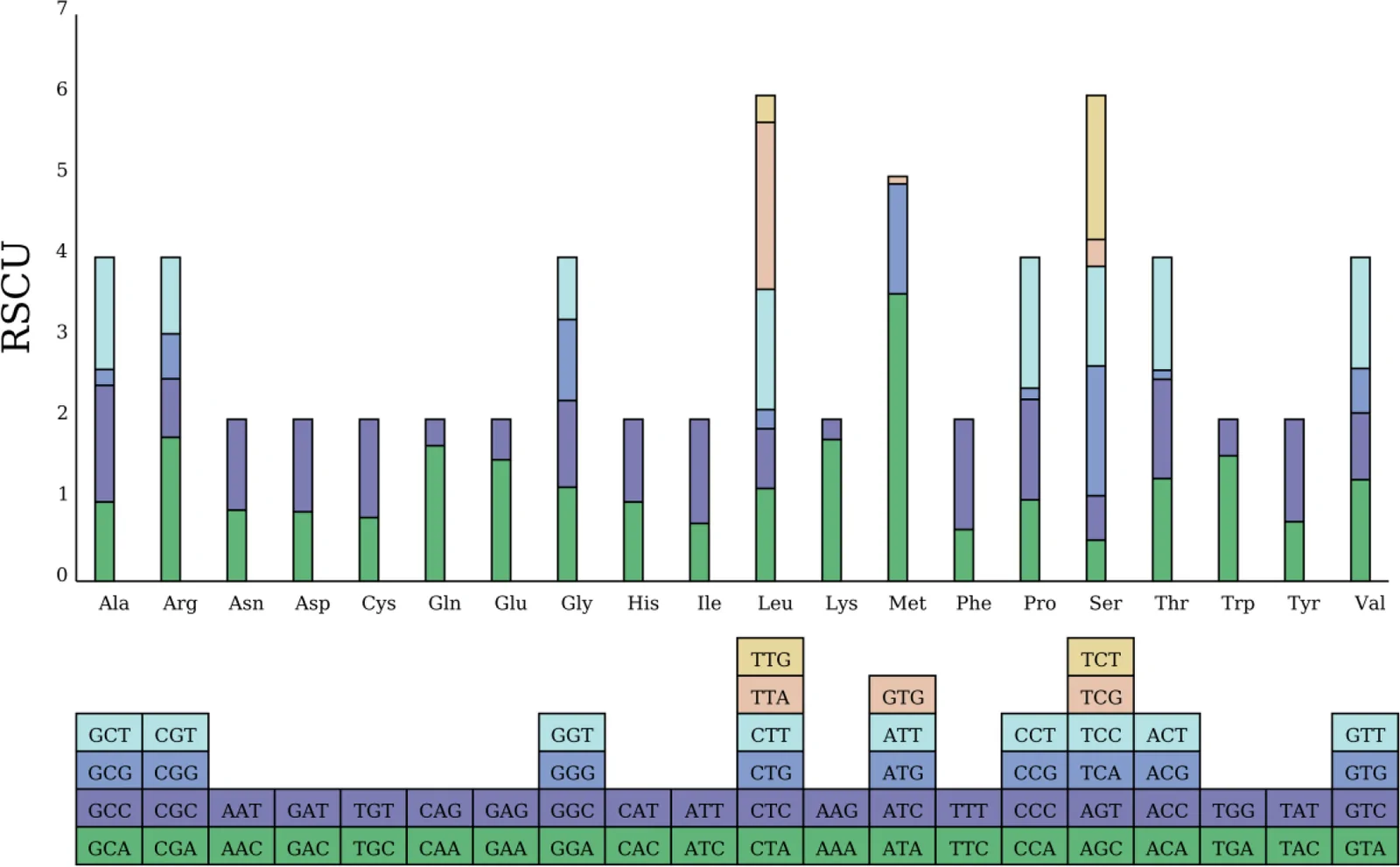
Relative synonymous codon usage (RSCU) of mitochondrial protein-coding genes in *Scutiger boulengeri*. Codon families are shown below the histogram, and bar heights represent the cumulative RSCU values of synonymous codons.

**Table 5.** Codon counts and relative synonymous codon usage (RSCU) values of the 13 mitochondrial protein-coding genes of *Scutiger boulengeri*.

**Codon**	**No**.	**RSCU**	**Codon**	**No**.	**RSCU**	**Codon**	**No**.	**RSCU**
AGA(*)	0	0	AUU(I)	211	1.2866	CGA(R)	32	1.7776
AGG(*)	1	0.3076	AAA(K)	70	1.75	CGC(R)	13	0.7224
UAA(*)	11	3.3848	AAG(K)	10	0.25	CGG(R)	10	0.5556
UAG(*)	1	0.3076	CUA(L)	121	1.1454	CGU(R)	17	0.9444
GCA(A)	70	0.9792	CUC(L)	78	0.738	AGC(S)	26	0.5082
GCC(A)	103	1.4404	CUG(L)	25	0.2364	AGU(S)	28	0.5472
GCG(A)	14	0.196	CUU(L)	157	1.4856	UCA(S)	82	1.6026
GCU(A)	99	1.3848	UUA(L)	218	2.0628	UCC(S)	63	1.2312
UGC(C)	11	0.7858	UUG(L)	35	0.3312	UCG(S)	17	0.3324
UGU(C)	17	1.2142	AUA(M)	115	3.5495	UCU(S)	91	1.7784
GAC(D)	30	0.8572	AUC(M)	0	0	ACA(T)	91	1.2684
GAU(D)	40	1.1428	AUG(M)	44	1.358	ACC(T)	88	1.2264
GAA(E)	69	1.5	AUU(M)	0	0	ACG(T)	8	0.1116
GAG(E)	23	0.5	GUG(M)	3	0.0925	ACU(T)	100	1.3936
UUC(F)	85	0.639	AAC(N)	58	0.8788	GUA(V)	64	1.2548
UUU(F)	181	1.361	AAU(N)	74	1.1212	GUC(V)	42	0.8236
GGA(G)	65	1.1608	CCA(P)	51	1.0048	GUG(V)	28	0.5492
GGC(G)	60	1.0716	CCC(P)	63	1.2412	GUU(V)	70	1.3724
GGG(G)	56	1	CCG(P)	7	0.138	UGA(W)	86	1.5496
GGU(G)	43	0.768	CCU(P)	82	1.6156	UGG(W)	25	0.4504
CAC(H)	47	0.9792	CAA(Q)	77	1.674	UAC(Y)	43	0.735
CAU(H)	49	1.0208	CAG(Q)	15	0.326	UAU(Y)	74	1.265
AUC(I)	117	0.7134						

### tRNA and rRNA genes, and putative control region

A total of 22 tRNA genes were identified in the mitochondrial genome of *S. boulengeri*. The combined length of these tRNAs was 1,540 bp, accounting for ~ 3.1% of the total mitochondrial genome, with individual tRNA genes ranging from 63 to 76 bp in length (Table 4). The tRNA region exhibited an *AT-skew* value of 0.017 (Table 3). Secondary structure predictions showed that all tRNAs could be folded into the canonical cloverleaf secondary structure except for *trnS2*, which lacked the DHU arm and instead formed a loop structure (Fig. 5). A total of 18 non-canonical base pairings were identified within the predicted secondary structures, including 17 G-U pairs and one U-U pair, predominantly distributed in the DHU and TΨC arms. The mitochondrial genome contains two ribosomal RNA (rRNA) genes, namely *rrnS* (12S rRNA) and *rrnL* (16S rRNA), with lengths of 930 bp and 1,600 bp, respectively. *rrnS* is located between *trnF* and *trnV*, whereas *rrnL* is located between *trnV* and *trnL2* (Table 4). The expanded non-coding region encompassing the putative mitochondrial control region is located downstream of *trnQ* and extends to the end of the mitochondrial genome, with a length of 22,418 bp (Table 4). This region exhibited an A+T content of 74.70%, with *AT-skew* and *GC-skew* values of –0.089 and –0.337, respectively (Table 3), indicating a pronounced A+T-rich composition.

**Figure 5.**
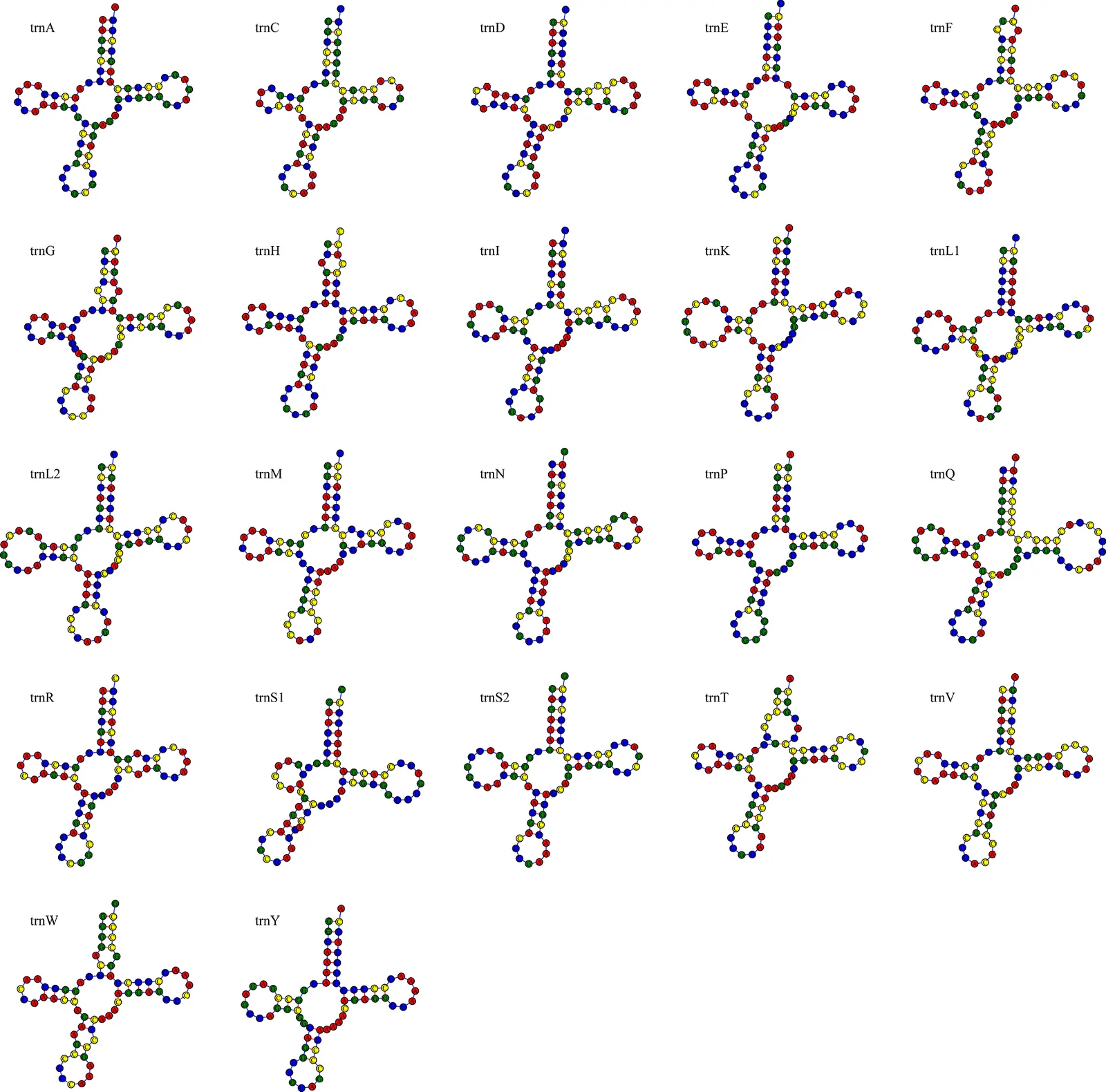
Predicted secondary structures of the 22 mitochondrial transfer RNA (tRNA) genes of *Scutiger boulengeri*. Except for *trnS2*, which lacks the DHU arm, all tRNAs exhibit the typical cloverleaf secondary structure. Non-canonical base pairings are mainly distributed within the DHU and TΨC arms.

### Phylogenetic analysis

Phylogenetic trees reconstructed based on *cytb* sequences of *Scutiger* and related taxa revealed highly congruent topologies between BI and ML analyses (Fig. 6). *Scutiger boulengeri* sequences from diverse sources did not form a mutually monophyletic clade; instead, several *S. boulengeri* individuals clustered closely with species such as *S. liupanensis* and *S. jiulongensis*. Overall, *cytb* dataset supported close maternal relationship between *S. boulengeri* and *S. liupanensis*.

**Figure 6.**
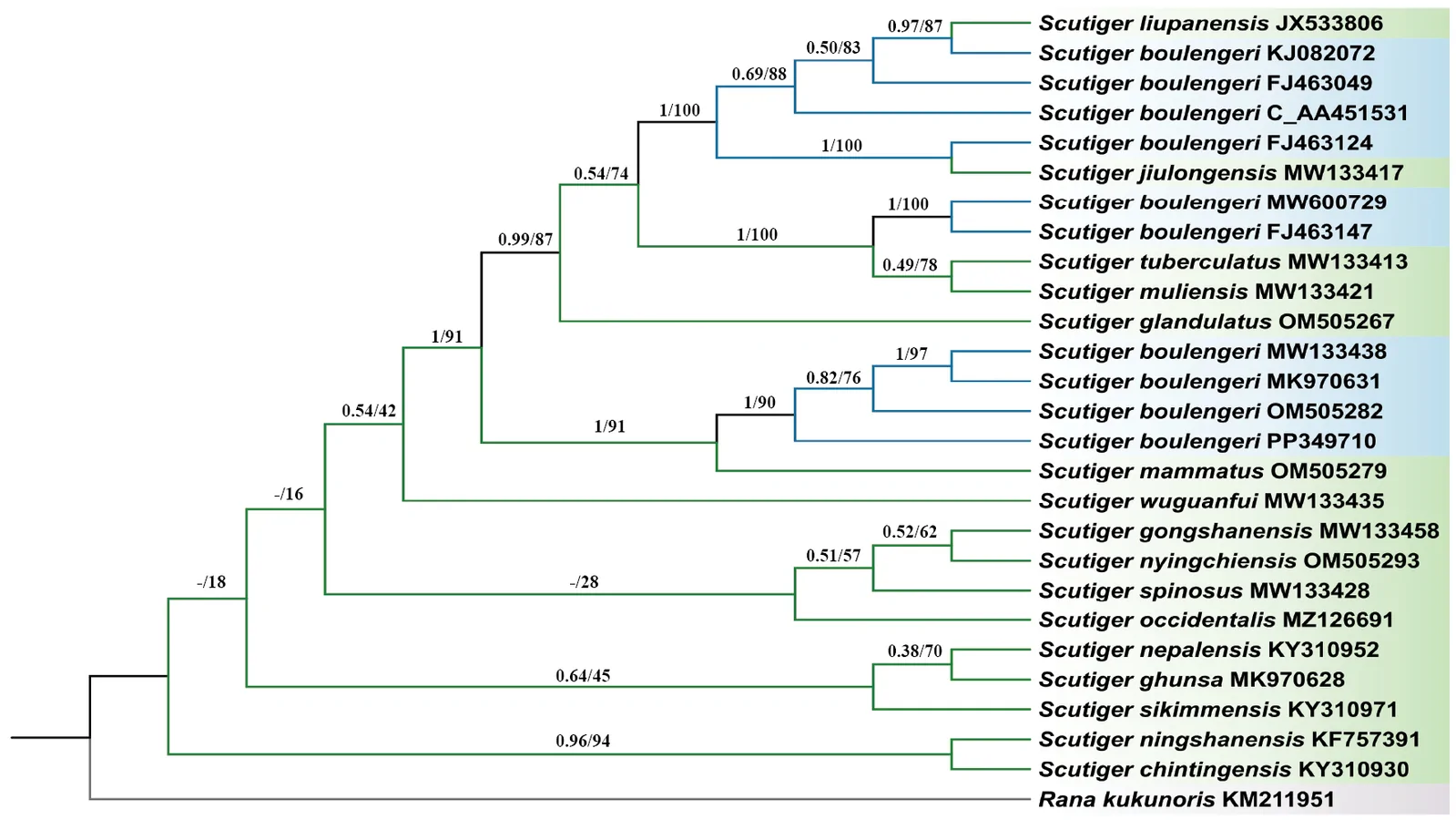
Phylogenetic relationships of *Scutiger* and related taxa inferred from mitochondrial *cytb* sequences using Bayesian inference (BI) and Maximum likelihood (ML) analyses. Values at the nodes indicate Bayesian posterior probabilities (PP) and ML bootstrap support values (BS), respectively. *Rana kukunoris* was used as the outgroup.

Phylogenetic trees reconstructed from concatenated sequences of 13 PCGs showed that all major genera within Megophryidae formed distinct clades with high support values (Fig. 7). Specifically, the genus *Scutiger* was recovered as monophyletic, in which *S. boulengeri* and *S. liupanensis* formed a sister-group relationship (BI = 1.00, BS = 99) and subsequently clustered with *S. ningshanensis*. Compared to cytb single-gene tree, phylogenetic tree reconstructed from 13 PCGs dataset yielded higher node support values and clearer topological resolution.

**Figure 7.**
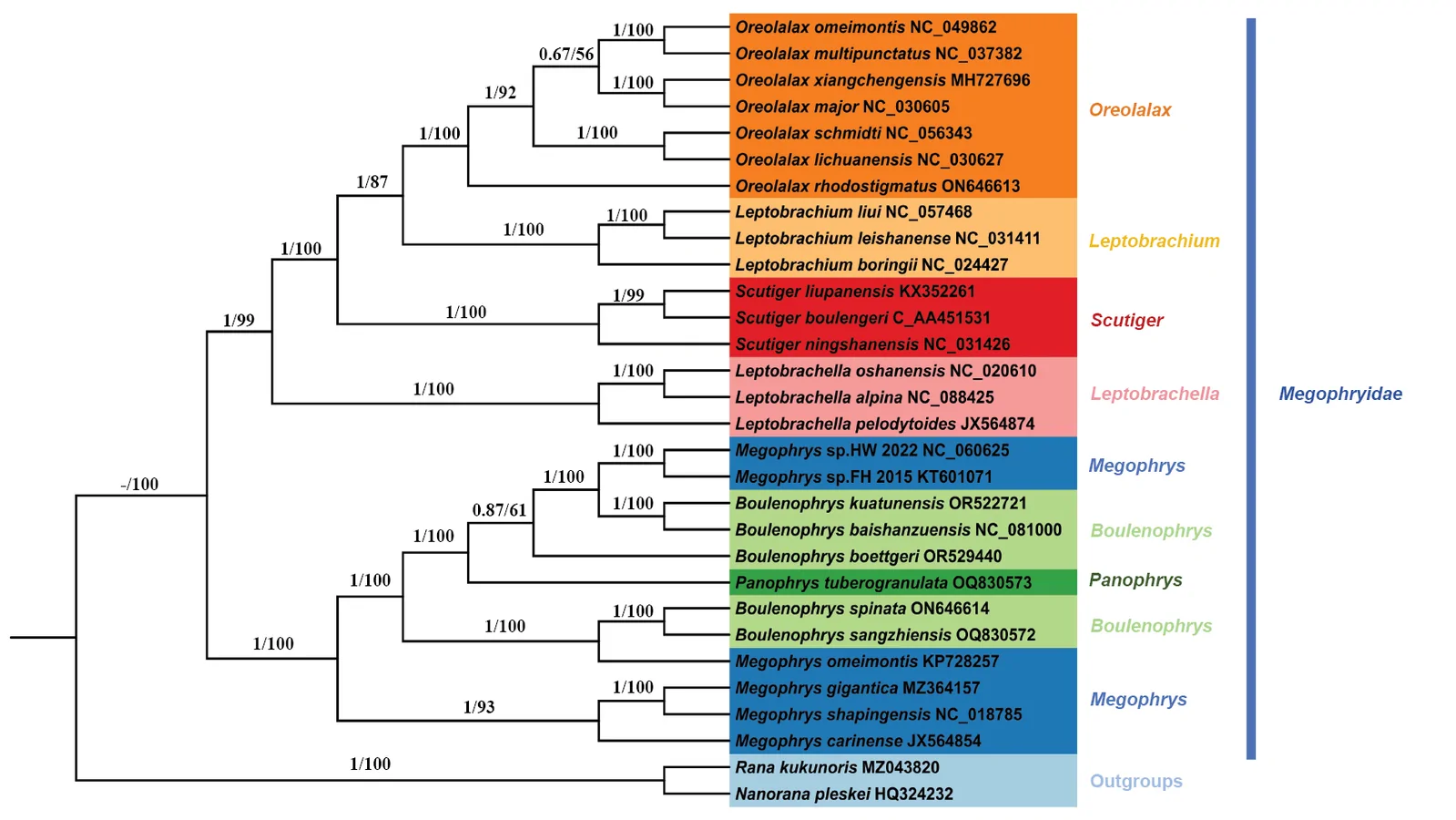
Phylogenetic relationships within Megophryidae inferred from the concatenated dataset of 13 mitochondrial protein-coding genes using Bayesian inference (BI) and maximum likelihood (ML) analyses. Values at the nodes indicate Bayesian posterior probabilities (PP) and ML bootstrap support values (BS), respectively. *Rana kukunoris* and *Nanorana pleskei* were designated as outgroups.

### Selection pressure analysis of *Scutiger* species

Ka/Ks analysis of 13 PCGs in *S. boulengeri* revealed variation in Ka/Ks ratios among different genes; however, the ratios for all genes were consistently below 1 (Fig. 8). Specifically, *atp8* exhibited a relatively high Ka/Ks ratio, whereas remaining genes remained at lower levels. Overall, low levels of non-synonymous substitutions were observed across all 13 PCGs, indicating that mitochondrial protein-coding genes of *S. boulengeri* are collectively under strong purifying selection constraints.

**Figure 8.**
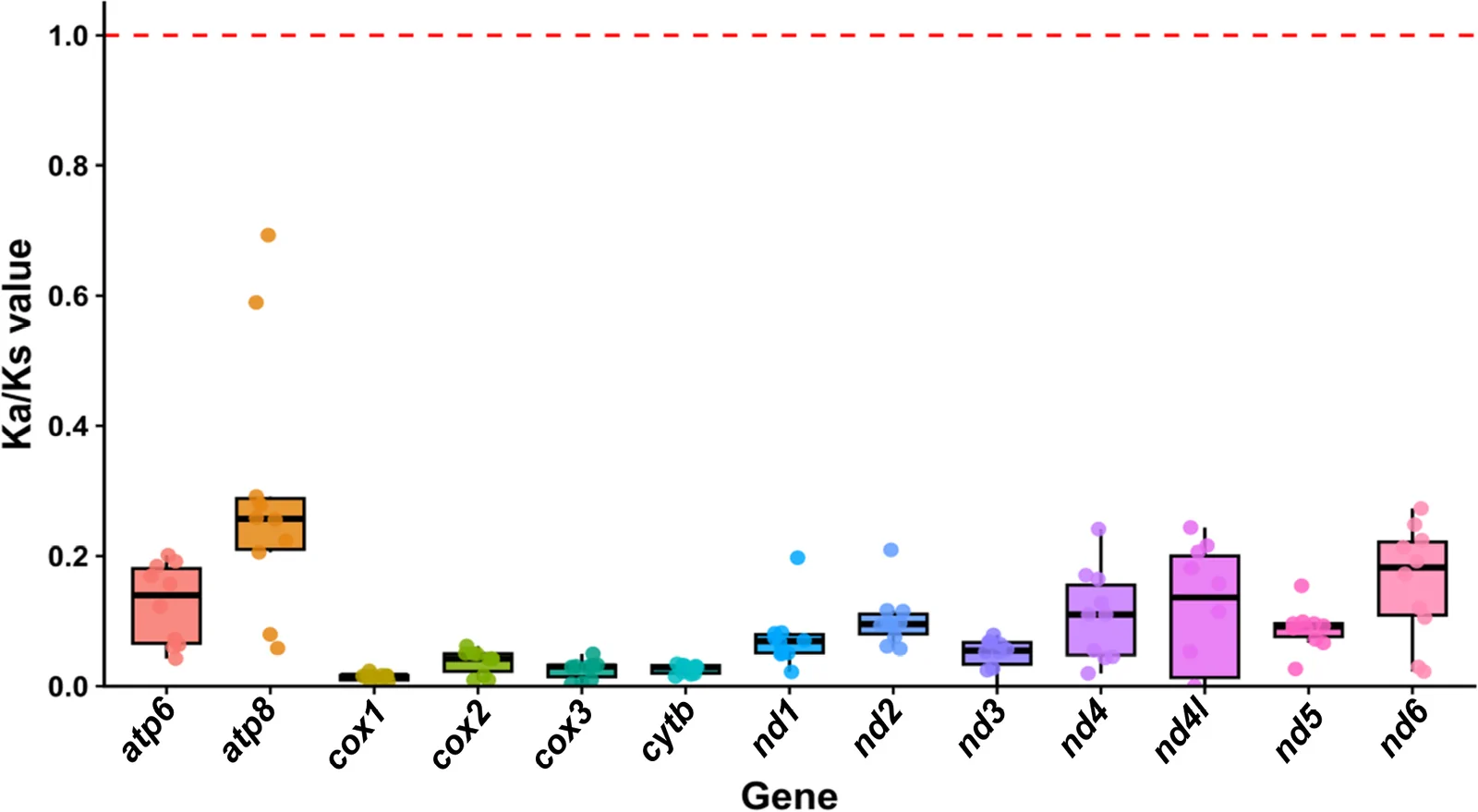
Selection pressure analysis of the 13 mitochondrial protein-coding genes of *Scutiger boulengeri*. Ka/Ks ratios were estimated under the Yang-Nielsen model. The dashed horizontal line indicates the neutral expectation (Ka/Ks = 1).

### Divergence time estimation of *Scutiger* and related lineages

Divergence time tree constructed based on the 13 PCGs dataset is presented in Fig. 9. The results show that divergence time between *Leptobrachella* and the clade comprising *Leptobrachium*, *Oreolalax*, and *Scutiger* was estimated at ~ 29.97 MYA; divergence time between *Leptobrachium* and the clade comprising *Oreolalax* and *Scutiger* was estimated at ~ 18.73 MYA. Within the genus *Scutiger*, divergence time between *S. ningshanensis* and the clade composed of *S. boulengeri* and *S. liupanensis* was estimated at ~ 5.96 MYA (95% HPD: 3.29–8.87 MYA). whereas divergence time between *S. boulengeri* and *S. liupanensis* was estimated at ~ 2.10 MYA (95% HPD: 1.11–3.32 MYA).

**Figure 9.**
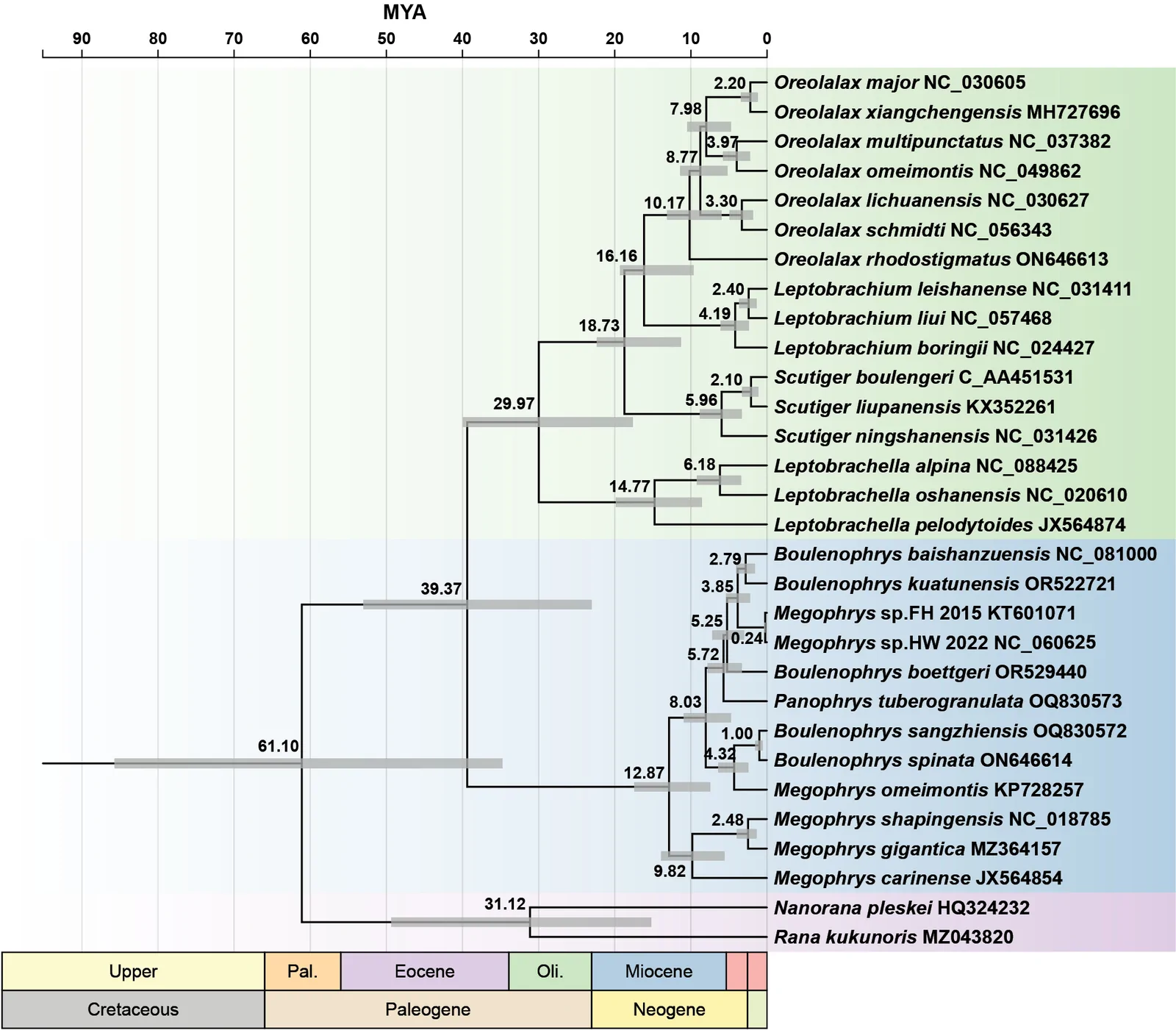
Divergence-time estimates inferred from the concatenated dataset of 13 PCGs. Numbers at the nodes indicate estimated divergence times (MYA), and horizontal bars represent the corresponding 95% highest posterior density (HPD) intervals.

## Discussion

### Structural characteristics of the *Scutiger boulengeri* mitochondrial genome and formation mechanism of expanded non-coding regions

The complete mitochondrial genome of *Scutiger boulengeri* obtained in this study is 49,991 bp in length, containing 13 protein-coding genes (PCGs), 22 tRNA genes, two rRNA genes, and an expanded non-coding region encompassing the putative mitochondrial control region. Although its gene composition is highly consistent with the typical structure of vertebrate mitochondrial genomes, its total length is significantly longer than those reported for the closely related species *S. liupanensis* and *S. ningshanensis*. Because the number of functional genes and the length of coding regions remain unchanged, the length increase of the *S. boulengeri* mitochondrial genome is primarily attributed to the expansion of non-coding regions. In contrast, the mitochondrial genome lengths of *S. ningshanensis* (16,799 bp and 17,282 bp) and *S. liupanensis* (16,890 bp) are maintained within the range typical for vertebrates (Fan 2016; Shan et al. 2025), further demonstrating that genome expansion in *S. boulengeri* is primarily driven by a substantial increase in non-coding regions. The A+T content of the *S. boulengeri* mitochondrial genome is 69.77%, exhibiting a strong AT bias. A high A+T content (61.05%) is also shared by *S. ningshanensis* (Shan et al. 2025), suggesting that AT enrichment may be a shared base composition characteristic of mitochondrial genomes within the genus *Scutiger*. Consequently, the primary differentiation between *S. boulengeri* and its close relatives does not stem from variation in base composition, but is instead concentrated within the expanded non-coding region encompassing the putative control region.

In this study, the expanded non-coding region encompassing the putative mitochondrial control region in *S. boulengeri* was found to reach 22,418 bp, representing the primary region responsible for marked expansion of the mitochondrial genome. Multiple tandem repeat units are present within this region, suggesting that accumulation of repetitive sequences may have contributed to its expansion process. In contrast, total length of 13 PCGs is 11,399 bp, and lengths of tRNA and rRNA regions are 1,540 bp and 2,530 bp, respectively, none of which exhibit substantial expansion alongside increase in overall genome size. Additionally, 16 intergenic spacers were identified in this genome with a combined length of 11,239 bp, the largest of which occurs between *trnW* and *trnF*, reaching 7,120 bp. Previous studies have demonstrated that mitochondrial genomes of *S. ningshanensis* and *S. liupanensis* are compact, with total intergenic spacer lengths of only 17 bp and 63 bp, respectively (Fan 2016). Therefore, increase in mitochondrial genome size in *S. boulengeri* is primarily associated with expansion of non-coding region encompassing putative control region and enlargement of adjacent intergenic spacers, rather than changes in functional genomic components.

Similar non-coding region expansions have also been documented in other animal taxa. For instance, studies on fish mitochondrial genomes revealed that control region and its flanking areas are hotspots for structural variation (Satoh et al. 2016); control region duplications and associated structural changes have also been reported in certain birds (Eberhard et al. 2001). Among amphibians, repetitive sequence expansion is likewise considered to be associated with mitochondrial genome size variation. For example, duplicated structures containing terminal portion of *cytb*, *trnT*, and non-coding regions have been identified in Tylototriton, the formation of which may have contributed to mitochondrial genome structural expansion (Kurabayashi et al. 2012).

Furthermore, in *S. boulengeri*, variations in the expanded non-coding region encompassing the putative control region, the long *trnW*-*trnF* spacer, and the adjacent region of *trnP*-*trnQ* are all concentrated within closely located genomic regions. Comparative studies of amphibian mitochondrial genomes have further demonstrated that the control region and some tRNA-associated regions represent hotspots of structural variation, and related rearrangement events may be associated with mechanisms such as tandem duplication-random loss (TDRL) (Zhang et al. 2021). In this study, we observed the translocation of *trnF* in the mitochondrial genome of *S. boulengeri*, accompanied by expansion of the *trnW*-*trnF* spacer and amplification of the adjacent non-coding region, suggesting that this region may have a high propensity for structural variation. Previous studies have reported that the control region of *S. ningshanensis* contains tandem repeats and insertion-deletion variations, and suggested that these structural changes may contribute to differences in mitochondrial genome length (Shan et al. 2025). Meanwhile, comparative analyses of amphibian mitochondrial genomes have shown that the control region and some tRNA-associated regions are relatively active sites of structural variation (Zhang et al. 2021). Taken together, the ultra-long non-coding region, expanded intergenic spacers, and relatively conserved functional gene composition of the *S. boulengeri* mitochondrial genome collectively indicate that its genomic expansion is primarily associated with the amplification of the non-coding region encompassing the putative control region and may involve the accumulation of repetitive sequences, replication slippage, and subsequent evolutionary processes of repetitive sequences (Asaeda et al. 2023).

### Conservation, codon usage bias, and selective pressure of protein-coding genes

For *S. boulengeri*, the Ka/Ks ratios of all 13 PCGs were below 1, indicating that the protein-coding regions as a whole are subject to purifying selection. Non-synonymous substitutions are constrained within the coding regions, demonstrating strong functional constraints on genes associated with mitochondrial oxidative phosphorylation during evolution. Shan et al. (2025) showed that in *S. ningshanensis*, all PCGs except *atp8* exhibited Ka/Ks < 1, further indicating that mitochondrial coding regions across the genus *Scutiger* are collectively under selective constraint. RSCU analysis revealed that codons such as UUA, AUA, UCU, and AAA showed higher usage frequencies in the PCGs of *S. boulengeri*, whereas codons like ACG, CCG, and GUG exhibited lower usage frequencies. Most of these high-frequency codons end in A or T, suggesting that codon usage bias may be associated with the AT bias of the mitochondrial genome. Zhang et al. (2021) noted in a comparative study of amphibian mitochondrial genomes that codon usage patterns are correlated with base composition. Barbhuiya et al. (2021) further pointed out that codon usage bias in amphibian mitochondrial genes may be jointly shaped by mutation pressure and natural selection. Therefore, the codon usage pattern of *S. boulengeri*PCGs likely reflects the combined effects of base composition bias and selective pressure.

In *S. boulengeri*, the A+T content of the PCG regions is 60.6%, whereas that of the expanded non-coding region encompassing the putative mitochondrial control region reaches 74.7%, demonstrating varying degrees of AT enrichment between the coding and expanded non-coding regions. Most PCGs begin with ATG, of which *cox1*, *atp8*, and *nad6* start with GTG; *cox1*, *cox2*, *cox3*, *nad3*, *nad4*, and *cytb* utilize T-- as an incomplete stop codon, while atp6 utilizes TA- as an incomplete stop codon. A study on *S. ningshanensis* and *S. liupanensis* by Fan (2016) likewise found that *atp8*, *cox1*, and *nad6* can use GTG as the start codon, and incomplete stop codons were also present. Temperley et al. (2010) indicated that incomplete stop codons in mitochondrial mRNAs can be completed into functional stop signals via post-transcriptional polyadenylation.

Differences in Ka/Ks ratios among various PCGs indicate that all 13 PCGs are constrained by purifying selection, although intensity of selective pressure may vary. Analysis of *Leptobrachium* mitochondrial genomes by Zhou et al. (2023) revealed differences in nucleotide diversity among different PCGs, showing that genes such as *atp8*, *atp6*, and *nad2* exhibited higher levels of variation, whereas *cox1*, *cox3*, and *cytb* were relatively conserved. For *S. boulengeri*, no PCGs with Ka/Ks > 1 were detected, indicating that its mitochondrial coding regions overall are in evolutionary state dominated by purifying selection. Because mitochondrial protein-coding genes are involved in oxidative phosphorylation, their low levels of non-synonymous substitution are likely associated with maintaining functional integrity of the corresponding proteins.

### Phylogenetic position and taxonomic significance of *Scutiger boulengeri*

Phylogenetic trees constructed based on the *cytb* gene sequence and the concatenated 13 PCGs sequence both support a close phylogenetic relationship between *S. boulengeri* and *S. liupanensis*. Specifically, the 13 PCGs dataset further supports that *S. boulengeri* and *S. liupanensis* are sister groups, and together with *S. ningshanensis*, they form a closely related clade, indicating that the phylogenetic position of *S. boulengeri* at the mitochondrial level is relatively stable. Shan et al. (2025) showed in their study on the mitochondrial genome of *S. ningshanensis* that it also supports the monophyly of the genus *Scutiger*, and indicates a close relationship between *S. ningshanensis* and *S. liupanensis*. Phylogenetic trees constructed based on PCGs, rRNAs, and combined datasets likewise retrieved *S. ningshanensis* and *S. liupanensis* clustered as a clade (Fan 2016). In summary, within the scope of the current taxon sampling, *S. boulengeri*, *S. liupanensis*, and *S. ningshanensis* constitute a relatively stable and closely related clade within the genus *Scutiger*. Furthermore, the 13 PCGs dataset indicates that *Scutiger* has a close phylogenetic relationship with related genera such as *Oreolalax*, *Leptobrachium*, and *Leptobrachella*, which is highly consistent with the mitochondrial phylogenetic studies of Megophryidae.

The sister-group relationship between *S. boulengeri* and *S. liupanensis* provides mitochondrial molecular evidence for their close relationship. With *S. boulengeri* serving as the comparison subject, the species description of *S. liupanensis* indicated that the differences between the two species are mainly reflected in characteristics such as body size, lateral fringe of toes, and vertical elevational distribution (Fan 2016). The molecular topology displays their closest relationship, suggesting that these morphological differences exist between groups with close genetic relationships, which can provide a reference for studies on the morphological and genetic differentiation of closely related Scutiger taxa. Lin et al. (2021) found that *S. boulengeri* could be divided into six genetic lineages, with obvious genetic differentiation among different lineages accompanied by certain niche differences. Although a single complete mitochondrial genome sample cannot represent the intraspecific genetic structure of *S. boulengeri* across its entire distribution range, the sample is stably clustered into *Scutiger* in both datasets, supporting the species identification of the current sample and its phylogenetic position within the genus.

Chen et al. (2009) compared the phylogenetic results of *cytb* and multiple nuclear genes, finding that obvious conflicts between mitochondrial and nuclear gene trees exist among high-altitude related species of *Scutiger*, such as *S. boulengeri*, *S. glandulatus*, *S. mammatus*, and S. tuberculatus, and suggested that historical mitochondrial gene introgression might have been involved in this phenomenon. These studies indicate that complex genetic histories may exist among related taxa of *Scutiger*, and a single or few mitochondrial markers might be insufficient to fully resolve the relationships among closely related taxa of this genus. Compared to a single mitochondrial fragment, the concatenated 13 PCGs sequence can provide more phylogenetic information. The results of the present study show that *S. boulengeri* forms a stable closely related clade with *S. liupanensis* and *S. ningshanensis*, further enriching the mitochondrial phylogenetic data of related taxa in the genus *Scutiger*. This mitochondrial genome evidence can provide a foundation for future studies combining multiple geographical populations, nuclear genomic data, and morphological evidence to explore genetic differentiation and species delimitation of *S. boulengeri* and its related groups.

### Divergence time, geographical isolation, and high-altitude diversification of the genus *Scutiger*

Preliminary divergence time estimation based on the concatenated sequences of the 13 PCGs in this study indicates that divergence between *Leptobrachella* and the clade comprising *Leptobrachium*, *Oreolalax*, and *Scutiger* occurred at ~ 29.97 MYA. Divergence between *Leptobrachium* and the clade comprising *Oreolalax* and *Scutiger* occurred at ~ 18.73 MYA. Within the genus *Scutiger*, *S. ningshanensis* diverged from the clade formed by *S. boulengeri* and *S. liupanensis* at ~ 5.96 MYA (95% HPD: 3.29–8.87 MYA), while the divergence between *S. boulengeri* and *S. liupanensis* occurred at ~ 2.10 MYA (95% HPD: 1.11–3.32 MYA). This maternally inherited chronological framework suggests that formation of this closely related clade experienced a hierarchical divergence process, ranging from deep divergence during the Oligocene/Miocene to the establishment of closely related species during the Pliocene/Pleistocene. Although inferring phylogeny based on a single maternal marker has its limitations, these estimates provide a foundational temporal reference for understanding the lineage divergence background of *Scutiger* and its related taxa.

The two estimated deep nodes at 29.97 MYA and 18.73 MYA temporally overlap with significant uplift and enhanced environmental heterogeneity of the Qinghai-Xizang Plateau from the Oligocene to the Miocene. Hofmann et al. (2017) proposed that the Himalayan *Scutiger* taxa might have originated in Paleo-Tibet, and their early lineage divergence could be associated with Qinghai-Xizang Plateau uplift and orogeny. In this study, the topology showing early divergence of *S. ningshanensis* and formation of a closely related clade by *S. boulengeri* and *S. liupanensis* is largely consistent with the regional geographic divergence pattern defined by Shi et al. (2023). The divergence node of *S. boulengeri* and *S. liupanensis* at 2.10 MYA identified in this study postdates the main active period of the orogeny, coinciding with the intensification of early glacial cycles during the Quaternary (Pleistocene). This suggests that early main lineage divergence of this group may have been driven by tectonic uplift, whereas recent formation of closely related species within the genus was likely influenced more by drastic Pleistocene climate fluctuations and geographic isolation during glacial periods.

Geographic isolation and high-altitude environmental heterogeneity are likely key factors influencing divergence of the closely related *S. boulengeri* clade. Research by Wang et al. (2022) on the eastern margin of the Qinghai-Xizang Plateau demonstrated that environmental heterogeneity significantly affects distribution patterns and community structures of amphibians. Furthermore, the barrier effect of complex topography on gene flow is constrained by inherent dispersal abilities and ecological characteristics of the species (Sánchez-Montes et al. 2018). In the present study, expansion of the putative mitochondrial control region spanning up to 22 kb in *S. boulengeri* was revealed for the first time. Under severe high-altitude glacial pressures of the Pleistocene, this mitochondrial structural variation, driven by massive non-coding repetitive sequences, may represent a response to microenvironmental heterogeneity or specific metabolic requirements under extreme cold and hypoxic conditions. Although direct functional validation is currently lacking, this discovery offers a unique perspective for understanding how drastic Pleistocene climate changes impacted basal molecular evolution of plateau amphibians.

This study provides preliminary evolutionary timeline clues for *Scutiger* and its related taxa, suggesting that their formation is likely the combined result of Tertiary tectonic uplift and Quaternary climate fluctuations. Due to limitations of incomplete lineage sorting (ILS), recent historical gene flow, and maternal inheritance bias (Shan et al. 2025), relying solely on a single mitochondrial 13 PCG dataset has inevitable limitations in inferring recent species radiation. Therefore, Pleistocene and other divergence times estimated in this study should be treated as preliminary references. Rather than definitively clarifying phylogenetic relationships, the core contribution of this study lies in filling the gap of the complete mitochondrial genome of *S. boulengeri* for the first time and characterizing its abnormally expanded structural features. In the future, further studies incorporating multi-locus nuclear or whole-genome data, extensive population sampling, and precise fossil calibration models are urgently needed to ultimately disentangle the complex evolutionary network and high-altitude adaptation mechanisms of this genus.

## Conclusions

In this study, the circular mitochondrial genome of *Scutiger boulengeri*, measuring 49,991 bp in length, was sequenced and assembled using a combination of Illumina and Oxford Nanopore sequencing technologies. The genome contains 13 PCGs, 22 tRNA genes, two rRNA genes, and an expanded non-coding region putatively containing the mitochondrial control region, with an overall gene composition largely consistent with typical vertebrate mitochondrial genomes. Compared to most amphibian mitochondrial genomes, the genome of *S. boulengeri* exhibits a certain degree of expansion, which is likely primarily associated with the enlarged putative control region and its adjacent *trnW*-*trnF* and *trnP*-*trnQ* non-coding regions. The Ka/Ks values for all 13 PCGs are less than 1, suggesting that these genes may be subjected to purifying selection constraints. Phylogenetic analyses preliminarily suggest a close relationship between *S. boulengeri* and *S. liupanensis*, which together constitute a closely related clade with *S. ningshanensis*. Due to currently limited availability of mitochondrial genome data for the genus *Scutiger*, comprehensive understanding of genomic structural evolution and species relationships within this group remains constrained. Future studies incorporating multi-locus nuclear or whole-genome data, along with expanded taxon sampling of this genus and its related lineages, are necessary to further explore its phylogenetic position and complex evolutionary relationships.

## Additional information

### Artificial Intelligence (AI) use

The authors accept full responsibility for the content of the manuscript, including the disclosure of any use of AI.

This article uses ChatGPT (OpenAI) solely to refine the language of the manuscript and improve its readability.

### Author ORCIDs

X.-z. Zhang https://orcid.org/0000-0002-8429-2367

### Data availability

The complete mitochondrial genome of the species has been deposited in CNCB GenBase under accession number C_AA451531. All analyses and results in this study were based on this sequence and other sequences available in public databases.

## Supplementary materials

Supplementary material 1 Supplementary figures Data type zip Explanation note **fig. S1**. Coverage. **fig. S2**. Read mapping. **fig. S3**. Long-read coverage. **fig. S4**. Science and Technology Ethics. **fig. S5**. Convergence diagnostics. https://binary.pensoft.net/file/1788996This dataset is made available under the Open Database License (http://opendatacommons.org/licenses/odbl/1.0/). The Open Database License (ODbL) is a license agreement intended to allow users to freely share, modify, and use this Dataset while maintaining this same freedom for others, provided that the original source and author(s) are credited. Wan-ting Wang, Fu-hao Zhang, Rui Wang, Xu-ze Zhang

Supplementary material 2 TRF Data type xlsx https://binary.pensoft.net/file/1788997This dataset is made available under the Open Database License (http://opendatacommons.org/licenses/odbl/1.0/). The Open Database License (ODbL) is a license agreement intended to allow users to freely share, modify, and use this Dataset while maintaining this same freedom for others, provided that the original source and author(s) are credited. Wan-ting Wang, Fu-hao Zhang, Rui Wang, Xu-ze Zhang

Supplementary material 3 Blast Data type txt https://binary.pensoft.net/file/1788998This dataset is made available under the Open Database License (http://opendatacommons.org/licenses/odbl/1.0/). The Open Database License (ODbL) is a license agreement intended to allow users to freely share, modify, and use this Dataset while maintaining this same freedom for others, provided that the original source and author(s) are credited. Wan-ting Wang, Fu-hao Zhang, Rui Wang, Xu-ze Zhang
