# Supplementary material for: Mitochondrial genome of Scutiger boulengeri (Anura, Megophryidae) reveals an exceptionally expanded non-coding region and its phylogenetic position within Megophryidae
Source: Zookeys. 2026 Sep 24;1293:17–44. doi: 10.3897/zookeys.1293.203467 (PMC13631551; doi:10.3897/zookeys.1293.203467)
Supplement: Supplementary file 1 [file zookeys-1293-017_article-203467__-s001.zip › 203467_0R-2-A_Revised_Supplementary_Figure_4_Science_and_Technology_Ethics.pdf]

青海民族大学科技伦理审查申请表

申请时间：2025 年 12 月 30 日

<table>
<tr><td>序号
（由学校统一填写）</td><td colspan="3">2025-029</td><td>项目单位</td><td>生态环境与资源学院</td></tr>
<tr><td>项目名称</td><td colspan="3">高原林蛙低氧适应及强紫外环境适应的分子机制研究</td><td>项目起止时间</td><td>202312-202712</td></tr>
<tr><td>项目类别</td><td colspan="5">A.✓生命科学　B. 医学　C. 人工智能　D.其他（请注明）：________</td></tr>
<tr><td colspan="6">申请人（项目负责人）简要信息</td></tr>
<tr><td>姓名</td><td>张滑泽</td><td>性别</td><td>男</td><td>学历</td><td>硕士研究生</td></tr>
<tr><td>办公电话</td><td>0971-8801739</td><td>电子邮箱</td><td>zxz1904@126.com</td><td>移动电话</td><td>187973290632</td></tr>
<tr><td colspan="2">目前主要研究方向</td><td colspan="4">高原土著动物环境适应</td></tr>
<tr><td colspan="6">研究内容摘要：
本研究拟就高原林蛙接收环境信号，适应强紫外环境和低氧环境的机制开展研究， 对分布于不同海拔梯度的高原林蛙野外样本，系统测定形态结构特征及血液生理生化指标，分析高原林蛙环境适应的形态及生理特征；对其开展转录组、蛋白组和单细胞转录组等定量研究，对其基因及蛋白进行表达分析验证。</td></tr>
<tr><td colspan="6">申请人（项目负责人）承诺：
以上所填内容均属实，如获批准，我将严格按照提供的方案进行研究，并遵守青海民族大学科技伦理相关规定。
申请人（项目负责人）签字：张滑泽
日期：2025 年 12 月 30 日</td></tr>
<tr><td colspan="6">申报单位科研分管领导意见：
单位领导签字：闫世亮 31/12　　日期：2025.12.31
（印章：青海民族大学 生态环境与资源学院 6301012604528）</td></tr>
<tr><td colspan="6">学校审批意见：
☑同意　□修改后同意　□修改后重审　□不同意
（印章：青海民族大学 6301012610087）
日期：2025.12.31</td></tr>
</table>
